# A Rare Case of Cord Compression From Extra-Medullary Hematopoiesis (EMH) in Beta Thalassemia

**DOI:** 10.7759/cureus.38520

**Published:** 2023-05-04

**Authors:** Adina Amin, Natalia Crenesse-Cozien, Kalsi Amardeep, Revathy Sundaram

**Affiliations:** 1 Internal Medicine, NewYork-Presbyterian Brooklyn Methodist Hospital, Brooklyn, USA; 2 Neurology, Jackson Memorial Hospital, University of Miami Miller School of Medicine, Miami, USA; 3 Hematology and Oncology, New York-Presbyterian Brooklyn Methodist Hospital, Brooklyn, USA; 4 Hematology and Oncology, NewYork-Presbyterian Brooklyn Methodist Hospital, Brooklyn, USA

**Keywords:** transfusion-dependent thalassemia (tdt), hematopoiesis, spinal cord compression weakness, cord compression, hemosiderosis, extra-medullary hematopoiesis, beta –thalassemia major

## Abstract

Cord compression can arise from many different etiologies -- including trauma, degenerative changes, growths, neoplasms, or even abscesses. While some etiologies can cause symptoms such as weakness or motor deficits, others can simply present as pain. A rare cause of cord compression is extramedullary hematopoiesis (EMH), or the growth of blood cells outside the bone marrow. This rare, abnormal growth of cells can result in severe complications such as increased intracranial pressure and motor and sensory impairment. General clinicians should strive for early and prompt diagnosis of cord compression whenever possible, especially in patients who present with acute neurological deficits. We present a case of a 27-year-old female with beta thalassemia major (BTM) and transfusional hemosiderosis, who came in with progressive lower extremity weakness, numbness and urinary retention, and was diagnosed with acute cord compression from EMH.

## Introduction

Beta thalassemia major (BTM), also known as Cooley’s Anemia, is a rare, transfusion dependent, autosomal recessive disorder. It is caused by the reduced production of beta chains in the hemoglobin (Hb) molecule and an accumulation of excess alpha chains caused by a mutation in the HBB gene. There are several molecular defects which lead to this clinical expression of disease. Beta thalassemias can be divided into thalassemia major (TM) which is transfusion dependent, thalassemia intermedia (TI) which is non-transfusion-dependent, and thalassemia minor (or beta thalassemia trait) which is a carrier condition in which an individual is heterozygous for a beta+ or beta0 thalassemia mutation.. Those with transfusion dependence include severe forms of beta thalassemia (e.g. homozygous β0 thalassemia, β0/β+, β/δβ, and others) and require regular blood transfusions which are necessary for survival [1]. Beta thalassemia can lead to transfusional iron overload and thus cause complications ranging from short stature, to life threatening splenomegaly and cardiomyopathy. Extramedullary hematopoiesis (EMH), or the growth of blood cells outside the bone marrow, is a rare sequelae of BTM (<1%) [1]. It may result in hepatosplenomegaly, lymphadenopathy and pleural, pericardial, or abdominal effusions [2]. One severe complication of EMH is cord compression, which could result in motor and sensory impairment.

## Case presentation

**Figure 1:**
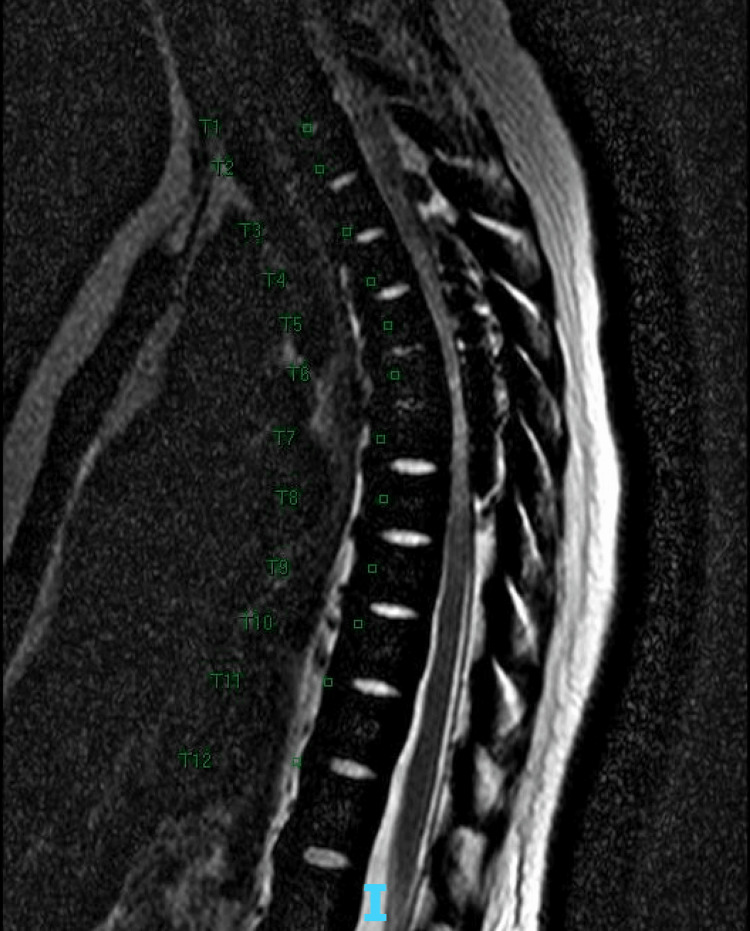
Initial thoracolumbar MRI showing severe cord compression from T3-T8 vertebrae.

We present a case of a 27-year-old female with BTM and transfusional hemosiderosis, admitted for fever and inability to ambulate. She was recently hospitalized eight days prior, with acute onset abdominal and back pain, and was found to have a ferritin level of 27,000 (mcg/L). She was treated with four doses of 2 g IV deferoxamine (chelation therapy), and was discharged home with instructions to follow up with her hematologist. Upon arrival to the emergency department the following week, she was noted to have numbness and weakness in bilateral lower extremities, as well as urinary retention. She was febrile to 38.4°C, tachycardic to 120 bpm, and had leukocytosis. Full neurological exam was performed which showed sensory deficits, weakness (5/5 strength in upper extremities, 2/5-3/5 strength in bilateral lower extremities), abnormal coordination and gait, as well as decreased sensation from the waist down, with a negative straight leg test. Blood work was significant for elevated liver enzymes (AST 312, ALT 341, ALP 142), likely due to the effect of hemosiderosis on the liver. She underwent a thoracolumbar spinal MRI which showed extensive soft tissue within the dorsal epidural spinal canal, mass effect from severe canal stenosis, as well as severe cord compression from T3-T8 vertebrae, as shown in Figure 1. 

She underwent emergent neurosurgical decompression, and a 4.7/3.1/1.4cm thoracic epidural mass with tan/red firm bone and tissue was excised and sent for pathology. Post procedure, she became hypotensive, and was admitted to the Neurology Intensive Care Unit (Neuro ICU) for blood pressure support and hourly neuro-checks. She required phenylephrine to maintain a mean arterial pressure greater than 85 bpm, (desired to improve spinal cord perfusion). 

**Figure 2:**
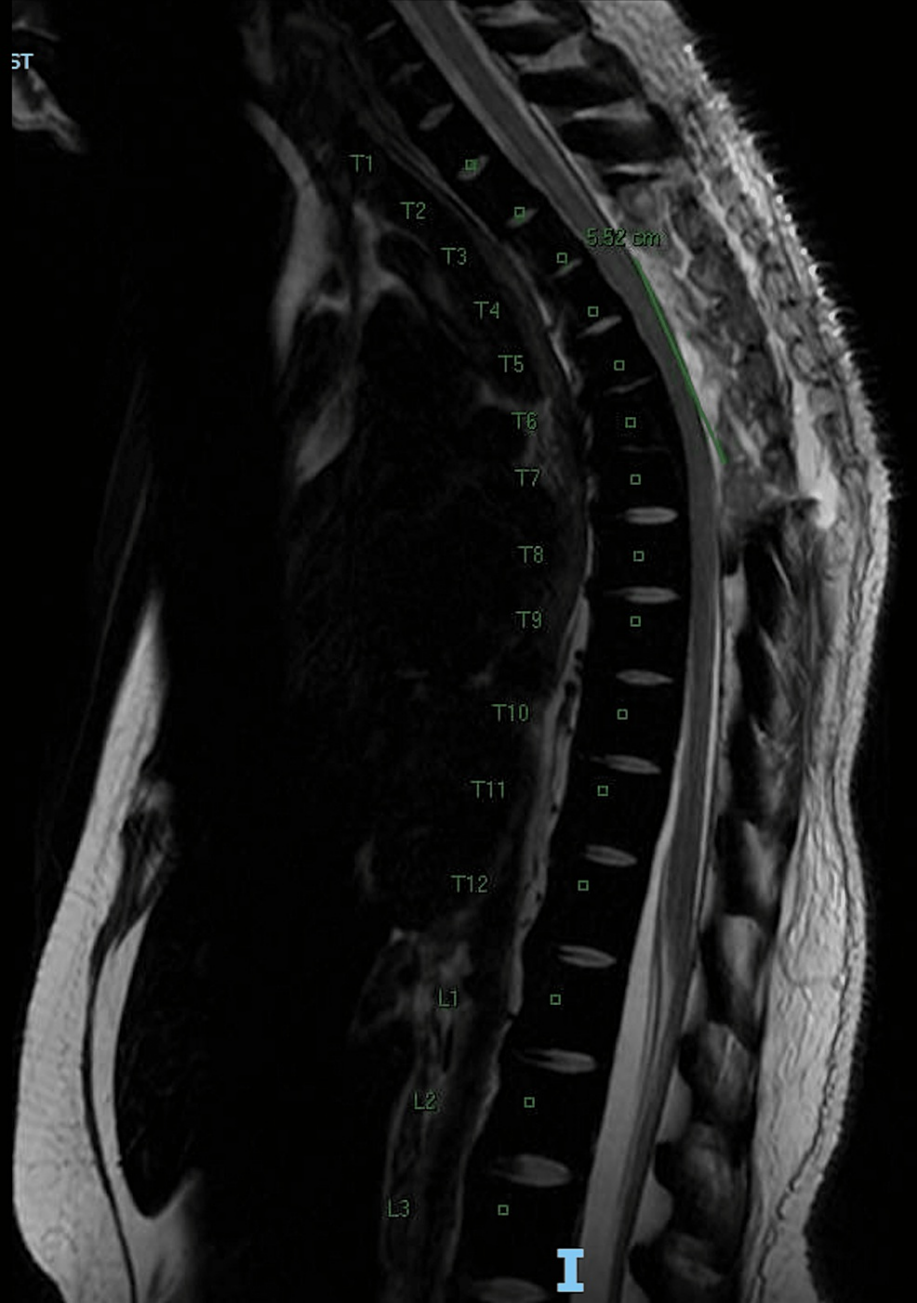
Thoracolumbar MRI post laminectomy.

Pathology results from her laminectomy showed extra-medullary hematopoiesis: erythroid precursors, granulocytes, megakaryocytes, and numerous hemosiderin-laden macrophages with occasional hemophagocytic histiocytes. A repeat MRI was obtained, which showed post-surgical changes of T3-T7 posterior decompression and resection of the upper thoracic dorsal epidural mass, with decreased mass effect/compression on the upper thoracic spinal cord and decreased associated spinal cord edema, as seen in Figure 2. It also showed a 1.1 cm rounded T2/T2 hypo-intense enhancing focus in dorsal space at the level of T8 which may represent residual lesion/extra-medullary hematopoiesis. 

Due to consistent rehabilitation and urgent removal of the inciting factor, our patient was eventually discharged home, with minimal residual deficits. She has fortunately made great strides towards recovery, progressively regaining motor function. She is currently on hydroxyurea as part of her treatment regimen, is completely asymptomatic and back at work.

## Discussion

Beta thalassemia is caused by reduced production of beta chains and accumulation of excess alpha chains, which may be due to variants that reduce the expression of beta globin (beta+) or completely eliminate beta globin expression [3]. There are several different sequelea that may result from BTM, with some of the most common being growth retardation, issues with fertility, and hypoadrenalism [4]. In BTM, chronic anemia leads to a constant hypoxic state, which results in increased erythropoietin (EPO) production. As EPO increases, JAK2 (Janus Kinase 2, a Protein Coding gene) is activated, resulting in increased ineffective extra-medullary erythropoiesis [5]. Hematopoiesis is the process by which the body produces blood cells, which can sometimes occur outside the bone marrow or extra-medullary. This is seen in infections, myeloproliferative neoplasms, lymphomas, leukemias, or as in this case, thalassemia. Organ involvement may present as splenomegaly, hepatomegaly, lymphadenopathy, or effusions. Patients can exhibit a variety of symptoms, including dysuria, respiratory distress, increased intracranial pressure, altered sensorium, motor and sensory impairment, including cord compression. EMH involvement in the spinal cord can present with symptoms such as back pain, paraparesis, or paresthesias, gait instability and possible urinary or fecal incontinence [3].

For patients with BTM, a goal pre-transfusion Hb of 9-10 (g/dL) could possibly help reduce the risk of EMH [6]. According to the 2016 AABB Red Blood Cell Guidelines, there is a recommended threshold for a HB of 7-8 g/dL, however, this does not apply to those with chronic transfusion dependent anemias, such as BTM [7]. The normal total body iron content in adults is 3-4 g, however, each unit of red blood cells contains 250 mg of iron [1]. Thus iron overload is a common concern for those with transfusion-dependent anemia. The nonspecific and gradual symptoms of iron overload can be easily misdiagnosed or overlooked. The most common causes of mortality in patients with transfusional hemosiderosis are cardiac related (cardiomyopathy and arrhythmias) [6]. Therefore, it is vital for these patients to obtain cardiac MRIs and have close monitoring of their cardiac function. 

Treatment for iron overload involves aggressive chelation therapy. Oral chelating agents are started early and monitored regularly. Transfusion regimens should also be optimized to maintain re-transfusion Hb between 9 and 10 (g/dL). In this patient, striking a balance between maintaining such a Hb goal while preventing iron overload was a challenge. Treatment options depend on timely diagnosis, and may include hydroxyurea, radiation, or surgical intervention.

## Conclusions

Extra-medullary hematopoiesis (EMH) leading to cord compression is a rare complication in transfusion-dependent BTM. It is usually found in patients with predisposing factors such as trauma or degenerative diseases. In those with delayed diagnosis, cord compression has a very high morbidity. Thus, early diagnosis and treatment of EMH as a spinal lesion is of utmost importance to avoid life threatening complications. A high index of suspicion should be maintained when any complaint of acute, persistent back pain is noted. Further research is warranted in order to establish factors contributing to EMH in the setting of BTM. Additionally, preventive regimens as well as treatment options need to be identified for those affected.
