# Correction: A Rare Case of Cord Compression From Extra-Medullary Hematopoiesis (EMH) in Beta Thalassemia

**DOI:** 10.7759/cureus.c113

**Published:** 2023-05-05

**Authors:** Adina Amin, Natalia Crenesse-Cozien, Kalsi Amardeep, Revathy Sundaram

**Affiliations:** 1 Internal Medicine, NewYork-Presbyterian Brooklyn Methodist Hospital, Brooklyn, USA; 2 Neurology, Jackson Memorial Hospital, University of Miami Miller School of Medicine, Miami, USA; 3 Hematology and Oncology, New York-Presbyterian Brooklyn Methodist Hospital, Brooklyn, USA; 4 Hematology and Oncology, NewYork-Presbyterian Brooklyn Methodist Hospital, Brooklyn, USA

This article has been corrected at the request of the submitting author to change a minor typo.

The last sentence of the introduction has been changed from “One severe complication of EMH is cord compression, which could result in including motor and sensory impairment” to “One severe complication of EMH is cord compression, which could result in motor and sensory impairment”.

The authors deeply regret that this error was not identified and addressed prior to publication.

 

